# Dietary isoleucine supplementation enhances growth performance, modulates the expression of genes related to amino acid transporters and protein metabolism, and gut microbiota in yellow-feathered chickens

**DOI:** 10.1016/j.psj.2023.102774

**Published:** 2023-05-09

**Authors:** D. Ruan, Q.L. Fan, S. Zhang, H.K. EI-Senousey, A.M. Fouad, X.J. Lin, X.L. Dong, Y.F. Deng, S.J. Yan, C.T. Zheng, Z.Y. Jiang, S.Q. Jiang

**Affiliations:** ⁎ Institute of Animal Science, Guangdong Academy of Agricultural Sciences, Key Laboratory of Animal Nutrition and Feed Science in South China, Ministry of Agriculture and Rural Affairs, State Key Laboratory of Livestock and Poultry Breeding, Guangdong Key Laboratory of Animal Breeding and Nutrition, Guangzhou 510640, China; † Department of Animal Production, Faculty of Agriculture, Cairo University, Giza 12613, Egypt; ‡ CJ International Trading Co., Ltd., Shanghai 201107, China; § Guangdong Key Laboratory for Crop Germplasm Resources Preservation and Utilization, Agro-biological Gene Research Center, Guangdong Academy of Agricultural Sciences, Guangzhou 510640, China

**Keywords:** isoleucine, yellow-feathered chickens, amino acid transporters, protein metabolism, gut microbiota

## Abstract

This study investigated the effects of dietary isoleucine (**Ile**) on growth performance, intestinal expression of amino acid transporters, protein metabolism-related genes and intestinal microbiota in starter phase Chinese yellow-feathered chickens. Female *Xinguang* yellow-feathered chickens (*n* = 1,080, aged 1 d) were randomly distributed to 6 treatments, each with 6 replicates of 30 birds. Chickens were fed diets with 6 levels of total Ile (6.8, 7.6, 8.4, 9.2, 10.0, and 10.8 g/kg) for 30 d. The average daily gain and feed conversion ratio were improved with dietary Ile levels (*P* < 0.05). Plasma uric acid content and glutamic-oxalacetic transaminase activity were linearly and quadratically decreased with increasing dietary Ile inclusion (*P* < 0.05). Dietary Ile level had a linear (*P* < 0.05) or quadratic (*P* < 0.05) effect on the jejunal expression of ribosomal protein S6 kinase B1 and eukaryotic translation initiation factor 4E binding protein 1. The relative expression of jejunal 20S proteasome subunit C2 and ileal muscle ring finger-containing protein 1 decreased linearly (*P* < 0.05) and quadratically (*P* < 0.05) with increasing dietary Ile levels. Dietary Ile level had a linear (*P* = 0.069) or quadratic (*P* < 0.05) effect on the gene expression of solute carrier family 15 member 1 in jejunum and solute carrier family 7 member 1 in ileum. In addition, bacterial 16S rDNA full-length sequencing showed that dietary Ile increased the cecal abundances of the Firmicutes phylum, and *Blautia, Lactobacillus*, and *unclassified_Lachnospiraceae* genera, while decreased that of Proteobacteria, *Alistipes*, and *Shigella*. Dietary Ile levels affected growth performance and modulated gut microbiota in yellow-feathered chickens. The appropriate level of dietary Ile can upregulate the expression of intestinal protein synthesis-related protein kinase genes and concomitantly inhibit the expression of proteolysis-related cathepsin genes.

## INTRODUCTION

Dietary amino acids are best known as building blocks for proteins and substrates for synthesis of small molecular substances and tissue growth, and have profound biological importance, crucial for whole body homeostasis (Eugenio et al., 2022). Among them, the branched-chain amino acids (**BCAA**), including isoleucine (**Ile**), leucine, and valine, in addition to precursor function, also play important roles in control of energy homeostasis, nutritional metabolism, immunity, and intestinal health in animals (Zhang et al., 2017; Nie et al., 2018; Brown et al., 2022, Kriseldi et al., 2022). It has been noted that the requirement for BCAA is influenced by the diet type, breed, and age of the birds. Several research strategies take advantages of low protein diets with BCAA supplementation to reduce cost and nitrogen excretion. Notably, few studies have explored the inclusion ratio of BCAA in a supplemental form and the effects on nutrients dynamic utilization under different disease challenges (Greenhalgh et al., 2022, Kim et al., 2022, Melare et al., 2019, Ospina-Rojas et al., 2020).

Isoleucine has been identified as the fourth or fifth limiting amino acid in the diet, depending on the feedstuffs used (Kidd et al., 2000, National Research Council, 1994). Ile and/or its derivatives improve growth performance, reproduction, meat quality, antioxidant capacity, regulate innate and adaptive immunity, and alleviate lipid deposition (Ren et al., 2016; Jiang et al., 2021; Lin et al., 2021; Wise et al., 2021; Mao et al., 2022; Oliveira et al., 2023). Protein metabolism consists of both proteolysis and protein synthesis, in part regulated by BCAA serving as signaling molecules via mechanistic target of the rapamycin (**MTOR**) pathway, important for muscle growth. Intestinal bacteria use dietary BCAA, and the abundance of some useful microbiota may be increased by BCAA. There are, however, few studies on BCAA focused in poultry (Kim et al., 2022). Yellow-feathered chickens account for almost 40% share of the broiler market in China, varying by strain or breed, price, and breeding cycles (FAS USDA, 2020). Species, age, and diet type all influence the avian need for BCAA. Chickens fed BCAA-deficient diets had lower protein accretion leading to growth and health issues (Zhang et al., 2017). It can be hypothesized that L-Ile is absolutely required for birds and they are regarded as dependent on the source used for dietary supplementation. The specific action in other animal studies of L-Ile stimulating protein synthesis has not been shown or established in poultry. Similarly, it is not clear whether L-Ile can improve amino acid transporter function. The BCAA in diet are also utilized by the intestinal microorganisms, and the BCAA are expected to enhance the abundance of some beneficial microbiota, but there have been few studies in poultry. The objective of present study was to investigate the effects of inclusion levels of dietary Ile on growth performance in starter phase yellow-feathered chickens; this included assessing gut microbiota structure and intestinal mucosal expression of amino acid transporters and protein metabolism, and estimation of the optimum requirements of dietary Ile.

## MATERIALS AND METHODS

### Birds, Experimental Diets, and Housing

This experiment was carried out in accordance with the standards set out by the European Union on Animal welfare and ethics, all of which were authorized by the Animal Ethics Committee of the Institute of Animal Science, Guangdong Academy of Agricultural Sciences (GAASISA-2021-052). A completely randomized block design with 6 graded contents of total Ile was used. The basal diet was formulated to satisfy nutritional requirements of medium-growing yellow chicks, with the exception of Ile (Table 1). The basal diet used wheat and peanut meal to achieve a low content of Ile (calculated 6.8 g/kg). The 5 additional treatments were the basal diet supplemented with 0.8, 1.6, 2.4, 3.2, and 4.0 g/kg L-Ile (90% purity, CJ CheilJedang Co., Ltd., Shanghai, China) making the total dietary Ile contents of 7.6, 8.4, 9.2, 10.0, and 10.8 g/kg of diet (calculated). Measured contents of Ile in hydrolyzes of the 6 mixed feeds, using amino acid analyzer (L-8900; Hitachi High-Technologies, Tokyo, Japan), were 6.8, 8.0, 8.6, 9.2, 10.0, and 10.6 g/kg of diet, respectively.

**Table 1:** Composition and nutrient content of the basal diet for yellow-feathered chickens (as-fed basis).

Component	Content, g/kg	Nutrient composition^2^	Level, g/kg
Corn	576.5	Metabolizable energy, MJ/kg	12.38
Wheat	50.0	CP	208.0
Soybean meal	202.0	EE	54.8
Peanut meal	60.0	Ca	9.1
Corn gluten meal	50.0	Total P	6.7
Soybean oil	15.0	Available P	4.1
L-Lysine·HCl (98.5%)	3.9	Total Lys	12.0
DL-Methionine (99%)	1.2	Total Met	4.4
L-Threonine hydrochloride (98.5%)	0.2	Total Met+Cys	7.9
L-Valine (98%)	1.0	Total Thr	8.5
Limestone	12.0	Total Trp	2.4
Dicalcium phosphate	15.2	Total Arg	12.3
Sodium chloride	3.0	Total Leu	18.8
Premix^1^	10.0	Total Val	9.9
Total	1000.0	Total Ile	6.8

1 The premix provided per kilogram of diet: vitamin A, 12,000 IU; vitamin D_3_, 600 IU; vitamin E, 45 mg; vitamin K_3_, 2.5 mg; vitamin B_1_, 2.4 mg; vitamin B_2_, 5.0 mg; vitamin B_6_, 2.8 mg; vitamin B_12_, 16 mg; choline, 1,300 mg; nicotinic acid, 42 mg; pantothenic acid, 12 mg; folic acid, 1.0 mg; biotin, 0.12 mg; Fe, 80 mg; Cu, 7.0 mg; Mn, 60 mg; Zn, 80 mg; I, 0.70 mg; Se, 0.15 mg.

2 Total Ile, Lys, Met, Met+Cys, Thr, Arg, Leu, Val, CP, EE, and Ca were means of triplicate measured values in the mixed feed. Other nutrient compositions are calculated values.

One thousand and eighty 1-day-old female *Xinguang* yellow-feathered chicks at initial BW 42.00 ± 0.06 g (Foshan Xinguang Agriculture and Animal Husbandry Co., Ltd., Foshan, China) were randomly assigned to the 6 dietary treatments, consisting of 180 birds per treatment, each containing 6 replicates. All replicates were housed in floor pens in an environmentally controlled room, and given ad libitum access to feed and water. A 5 cm depth of clean wood shaving litter was employed to cover the concrete floor pens.

### Broiler Performance

All chickens were weighed by pen, at the start and the end of 30 d experiment, and consumed feed was recorded on a per replicate basis. The average daily gain (**ADG**), average daily feed intake (**ADFI**), and feed conversion ratio (**FCR**; g feed/g BW gain) were calculated for the feeding phase. Mortality records were taken daily and the morality rate was calculated.

### Sample Collection

After 16-h feed withdrawal of 21 d, 2 birds per pen, approaching replicate average BW were selected. Heparinized blood samples were collected into 5 mL tubes and centrifuged at 3,000 × *r* at 4°C for 10 min. The plasma was collected and stored at −20°C until analysis. The birds were then electrically stunned and subsequently euthanized by exsanguination. Jejunal and ileal samples and cecal digesta were rapidly collected, frozen in liquid nitrogen, and then stored at −80°C until further analysis.

### Digestive and Immune Organ Indices

The proventriculus, muscular stomach, pancreas, duodenum, jejunum, ileum, bursa of Fabricius, thymus, and spleen were weighed and the indices of digestive organs and immune organs were calculated, based on live weight.

### Blood Biochemical Parameters Analysis

Plasma indices including the level of albumin (**ALB**), uric acid (**UA**), glucose (**GLU**), triglyceride (**TG**), cholesterol (**TC**), and activities of lactate dehydrogenase (**LDH**), creatine kinase (**CK**), glutamic-oxalacetic transaminase (**GOT**), and glutamic-pyruvate transaminase (**GPT**) were measured by the reagent kits (Nanjing Jiancheng Bioengineering Institute, Nanjing, China) according to the manual. The intra-assay CV was 2.3% and interassay CV was 5.34% for ALB; the intra-assay CV was 3.6% and interassay CV was 6.4% for UA and GLU; the intra-assay CV was 3.0% and interassay CV was 5.0% for TG; the intra-assay CV was 5.0% and interassay CV was 8.0% for TC; the intra-assay CV was 4.0% and interassay CV was 6.0% for LDH; the intra-assay CV was 2.3% and interassay CV was 5.3% for CK; the intra-assay CV was 5.0% and interassay CV was 7.0% for GOT; the intra-assay CV was 5.5% and interassay CV was 3.3% for GPT.

### Gene Expression Analysis

Total RNA was separated from each jejunal and ileal tissue sample with the use of TRIZOL reagent (Invitrogen, Carlsbad, CA). The RNA concentration was measured by a spectrophotometer (NanoDrop 1000, Thermo Fisher Scientific, Watertown, MA). RNA integrity was tested and confirmed by 1% agarose gel electrophoresis, only RNA samples with high quality (RNA integrity number ≥6.0) were used for transcription analysis. One microgram of total RNA was reverse transcribed into cDNA using a PrimeScript RT Regent Kit with gDNA Eraser (TaKaRa, Dalian, China). The primers used in this study shown in Table 2 were designed using Primer 5.0 version according to chicken (Gallus) sequences in GenBank and obtained from Sangong Biotech Co., Ltd. (Shanghai, China) and each gradient PCR products were tested and validated by 1% agarose gel electrophoresis and furtherly approved by real-time PCR.

**Table 2:** Primers of target genes used for quantitative real-time PCR.

Transcript^1^	Accession number	Primer sequence (5′–3′)	Product size (bp)	Annealing temperature (°C)
*MTOR*	XM_040689168.2	F: GGTGATGACCTTGCCAAACT R: CTCTTGTCATCGCAACCTCA	220	56
*RPS6KB1*	NM_001030721.2	F: GACCCAGTGACACTCCAGAA R: GTTATCCATGGGTGCTGCAG	176	57
*EIF4EBP1*	XM_424384	F: GCGAATGTAGGTGAAGAAGAG R: AACAGGAAGGCACTCAAGG	146	55
*MuRF1*	XM_424369	F: TGTCTATGGGCTGCAGAGGAA R: GGTGCTCCCCCTTCTTGAGT	230	60
*MAFbx*	NM_001030956.1	F: GACGCGCTTTCTCGATGAG R: CCTTGTTATTCAGTAGGTCTTTTTTCCT	152	55
*CC2*	AF027978.1	F: AACACACGCTGTTCTGGTTG R: CTGCGTTGGTATCTGGGTTT	241	56
*cathepsin B*	U18083.1	F: CAAGCTCAACACCACTGGAA R: TCAAAGGTATCCGGCAAATC	150	55
*SLC15A1*	XM_046906441.1	F: TAGACTGGGCAAGCGAGAAG R: AGCAGCAGCAACGAAAGC	344	60
*SLC1A1*	XM_424930.8	F: ACCCTTTTGCCTTGGAAACT R: TTGAGATGTTTGCGTGAAG	122	60
*SLC6A19*	XM_040663289.2	F: TCTGCCTGGGTTTGTCATCT R: AGCCAGTAATTGCCAGACCT	172	60
*SLC7A1*	XM_046908303.1	F: AGACATCTTCGCTGTGGTGA R: CGAGGATGTTGATGCAGGTG	107	57
*β-Actin*	NM_205518	F: GAGAAATTGTGCGTGACATCA R: CCTGAACCTCTCATTGCCA	152	55∼60

1 MTOR = mechanistic target of rapamycin; RPS6KB1 = ribosomal protein S6 kinase B1; EIF4EBP1 = eukaryotic translation initiation factor 4E binding protein 1; MuRF1 = muscle ring finger-containing protein 1; MAFbx = muscle atrophy F box protein; CC2 = 20S proteasome subunit C2; SLC15A1 = solute carrier family 15 member 1; SLC1A1 = solute carrier family 1 member 1; SLC6A19 = solute carrier family 6 member 19; SLC7A1 = solute carrier family 7 member 1.

The RT-PCR amplifications used and initial denaturation at 95°C for 30 s, followed by 40 cycles of 95°C for 15 s, annealing at *X*°C for 30 s and extension at 72°C for 30 s and were performed on a real-time PCR System (CFX 96, Bio-Rad, Hercules, CA). The reactions were carried out with 1 μL containing 200 ng of cDNA, 1 μL containing 10 pmol of each primer, and 7 μL nuclease-free water in an ultimate volume of 20 μL in accordance with the SYBER Green PCR Master Mix (TakaRa). Each target gene was run in duplicate, and the average of cycle threshold (**CT**) value was used to calculate 2^−∆∆^*^Ct^* for analysis of relative expression (Livak and Schmittgen, 2001). Data are shown with further normalization to values obtained from the basal diet.

### Full-Length 16S rRNA Sequencing of Gut Microbiota

The composition and structure of microbial communities from cecal digesta samples were analyzed using full-length (V1–V9) 16S rRNA high-throughput sequencing. Bacterial DNA was extracted using the E.Z.N.A. soil DNA Kit (Omega Bio-Tek, Norcross, GA) in accordance with the manual. The genomic DNA quality was verified by agarose gel electrophoresis. The full-length 16S rRNA gene was amplified with primer pairs 27F (5′-AGAGTTTGATCCTGGCTCAG-3′) and 1492R (5′-GTTACCTTGTTACGACTT-3′) using an ABI GeneAmp 9700 PCR thermocycler (Thermo Fisher Scientific). The detection of full-length 16S rRNA gene was sequenced on PacBio RS II platform (Majorbio Bio-Pharm Technology Co., Ltd., Shanghai, China). The raw reads were demultiplexed and quality-filtered using QIIME package to filter out high-quality sequences. The high-quality sequences were clustered into operational taxonomic units (**OTU**) at 97% similarity by UPARSE (version 7.1). The RDP Classifier (version 2.2) was used to annotate taxonomic information on each representative sequence. Alpha diversity indices, such as observed species, Chao1, ACE, Shannon, Simpson, and Good's Coverage, were assessed by Mothur (version v.1.30.1). Beta diversity was evaluated based on the unweighted and weighted unifrac distances between the OTU. Partial least squares discriminant analysis (**PLS-DA**) was performed using the R package mixOmics (https://www.mixOmics.org.) to reveal the variance of gut microbiota composition among treatments.

### Statistical Analysis

All data were presented as means, SEM or *P* values. Effects of dietary Ile were analyzed via 1-way ANOVA with GLM procedures of SAS (SAS Inst. Inc., Cary, NC). Duncan's multiple range test was used to compare discrepancies among the groups and *P* values <0.05 was considered significant. The orthogonal contrast analysis was performed to determine linear and quadratic effects of Ile. Quadratic regressions (*Y* = *c* + *bX* + *aX*^2^) and 2-slope broken line [*Y* = *L* + *U* × (*R* − *X*) (*X* < *R*); *Y* = *L* + *V* × (*X* − *R*) (*X* > *R*)] were used for the fixed response of the dependent variables to dietary Ile content as described by Dozier et al., (2009).

## RESULTS

### Growth Performance

The growth performance of chickens fed different dietary Ile is presented in Table 3. ADG, BW at 30 d of age and FCR showed significant linear and quadratic effects with increasing dietary inclusion of Ile (*P* < 0.05). There were no significant effects (*P* > 0.05) of increasing Ile on ADFI. Dietary Ile supplementation had no significant effects (*P* > 0.05) on digestive and immune organ indices of 30-day-old yellow-feathered chickens (Table S1).

**Table 3:** Effects of dietary L-isoleucine on the growth performance of yellow-feathered chickens in the starter phase.^1^

Indices^2^	Dietary Ile content, g/kg						SEM	*P* value		
	6.8	7.6	8.4	9.2	10.0	10.8		ANOVA	Linear	Quadratic
1 d BW (g)	42.02	42.05	42.03	42.00	42.06	42.04	0.015	0.272	0.549	0.385
30 d BW (g)	629.94^b^	674.29^a^	671.67^a^	671.14^a^	669.27^a^	667.74^a^	8.759	0.018	0.026	0.009
ADFI (g)	37.53	38.89	38.57	38.52	37.82	28.68	0.424	0.101	0.484	0.308
ADG (g)	19.60^b^	21.08^a^	20.99^a^	20.97^a^	20.91^a^	20.86^a^	0.292	0.019	0.026	0.009
FCR (g feed/g gain)	1.92^a^	1.85^b^	1.84^b^	1.84^b^	1.81^b^	1.86^b^	0.019	0.012	0.021	0.015
Mortality (%)	1.67	1.11	0.00	1.11	0.56	0.56	0.696	0.747	0.317	0.453

1 Means are from 30 birds per pen and 6 replicate pens per diet.

2 BW = body weight; ADFI = average daily feed intake; ADG = average daily gain; FCR = feed conversion ratio; NS = not significant.

a,b Means within a main effect with the same superscripts do not differ significantly (*P* < 0.05).

### Blood Biochemical Indices

In relation to the plasma parameters in the study (Table 4), dietary Ile supplementation significantly affected plasma ALB and UA content and GOT activity (*P* < 0.05). Plasma UA content and GOT activity were linearly and quadratically decreased with increasing dietary inclusion of isoleucine (*P* < 0.05). Dietary Ile supplementation had no significant effects (*P* > 0.05) on activities of LDH, CK, and GPT and contents of GLU, TG, and TC.

**Table 4:** Effects of dietary L-isoleucine on blood biochemical indexes of yellow-feathered chickens at 30 d of age.^1^

Indices^2^	Dietary Ile content, g/kg						SEM	*P* value		
	6.8	7.6	8.4	9.2	10.0	10.8		ANOVA	Linear	Quadratic
GLU, mmol/L	11.96	12.47	12.31	11.96	12.15	11.70	0.127	0.584	0.328	0.125
TG, mmol/L	0.37	0.35	0.39	0.34	0.35	0.35	0.010	0.765	0.406	0.691
TC, mmol/L	2.41	2.55	2.73	2.39	2.38	2.60	0.052	0.308	0.836	0.843
ALB, g/L	10.97^bc^	12.69^a^	12.22^ab^	10.54^c^	11.81abc	12.43^ab^	0.237	0.042	0.634	0.540
UA, mg/L	53.12^ab^	48.39^bc^	40.23^c^	41.93^bc^	52.79^ab^	61.10^a^	1.859	0.003	<0.001	<0.001
LDH, U/L	4148	4407	4031	4154	4038	4090	45.6	0.152	0.160	0.267
CK, U/mL	0.94	0.96	1.06	0.86	0.85	0.95	0.027	0.202	0.345	0.272
GOT, U/L	38.64^a^	37.52^ab^	36.88abc	23.78^d^	27.32^d^	28.54bcd	1.567	0.006	0.002	0.009
GPT, U/L	10.41	15.16	7.89	5.21	7.96	6.07	1.120	0.077	0.034	0.109

1 Means from 2 birds per pen and 6 replicate pens per diet.

2 GLU = glucose; TG = triglyceride; TC = cholesterol; ALB = albumin; UA = uric acid; LDH = lactate dehydrogenase; CK = creatine kinase; GPT = glutamic-pyruvate transaminase; GOT = glutamic-oxalacetic transaminase.

a–d Means within a main effect with the same superscripts do not differ significantly (*P* < 0.05).

### Expression of Genes Related to Protein Metabolism in the Jejunum and Ileum

**Figure 1:**
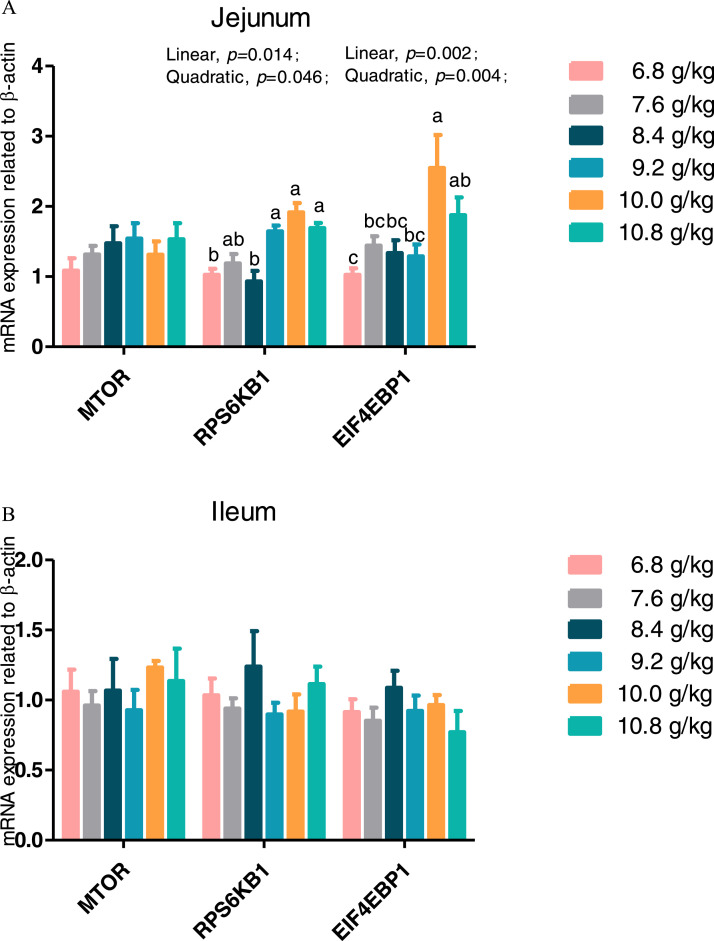
Effect of dietary L-isoleucine levels on the expression genes related to protein synthesis in jejunum and ileum. *n* = 6. (A) Relative mRNA abundances of mechanistic target of rapamycin (*MTOR*), ribosomal protein S6 kinase B1 (*RPS6KB1*), and eukaryotic translation initiation factor 4E binding protein 1 (*EIF4EBP1*) in jejunum. (B) Relative mRNA abundances of *MTOR, RPS6KB1*, and *EIF4EBP1* in ileum. Changes in mRNA abundances of *MTOR, RPS6KB1*, and *EIF4EBP1* were normalized to β-actin and expressed relative to the 6.8 g/kg L-isoleucine. Data are means ± SE, *n* = 6. Means with different letters (superscripts a, b) differ (*P* < 0.05).

**Figure 2:**
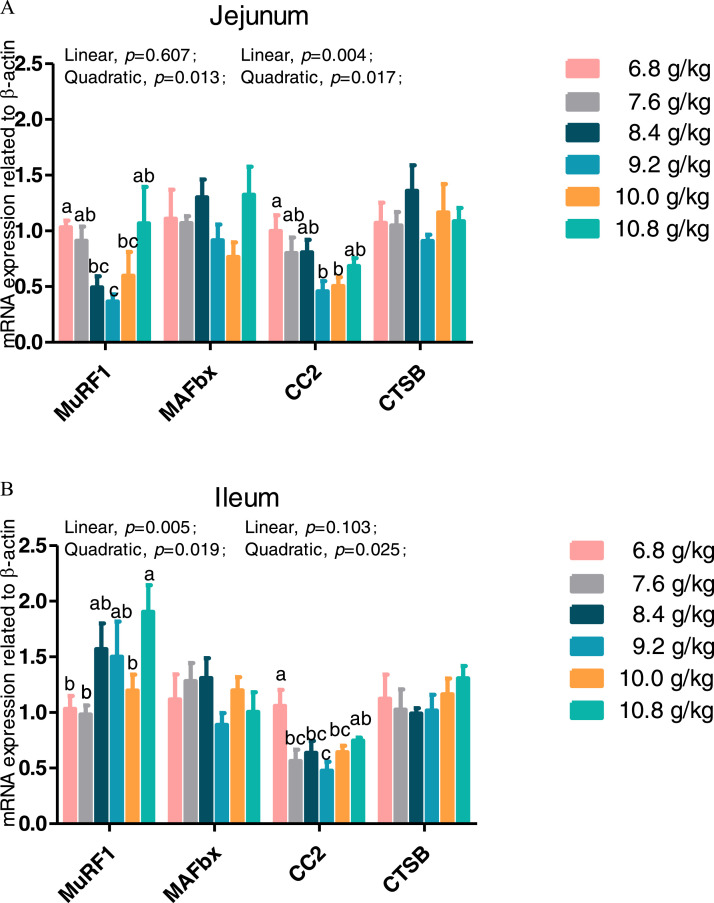
Effect of dietary L-isoleucine levels on the expression genes related to proteolysis in jejunum and ileum. *n* = 6. (A) Relative mRNA abundances of muscle ring finger-containing protein 1 (*MurF1*), muscle atrophy F box protein (*MAFbx*), 20S proteasome subunit C2 (*CC2*), and cathepsin B (*CTSB*) in jejunum. (B) Relative mRNA abundances of *MurF1, MAFbx, CC2*, and *CTSB* in ileum. Changes in mRNA abundances of *MurF1, MAFbx, CC2*, and *CTSB* were normalized to β-actin and expressed relative to the 6.8 g/kg L-isoleucine. Data are means ± SE, *n* = 6. Means with different letters (superscripts a, b) differ (*P* < 0.05).

As shown in Figures 1 and 2, the level of dietary Ile significantly affected (*P* < 0.05) the gene expression of ribosomal protein S6 kinase B1 (***RPS6KB1***), eukaryotic translation initiation factor 4E binding protein 1 (***EIF4EBP1***), muscle ring finger-containing protein 1 (***MuRF1***), and 20S proteasome subunit C2 (***CC2***) in mucosa of the jejunum and *MuRF1* and *CC2* in that of the ileum (*P* < 0.05). Expressions of *RPS6KB1* and *EIF4EBP1* in the jejunum showed a significant linear and quadratic increase (*P* < 0.05) with increased of dietary Ile, while jejunal and ileal *CC2* expression showed a significant linear and quadratic decreases (*P* < 0.05) with increased of dietary Ile. There were no significant effects of dietary Ile supplementation (*P* > 0.05) on the jejunal and ileal expression of *MTOR*, muscle atrophy F-box protein (***MAFbx***), and cathepsin B.

### Expression of Genes Related to Amino Acid Transporters in the Jejunum and Ileum

**Figure 3:**
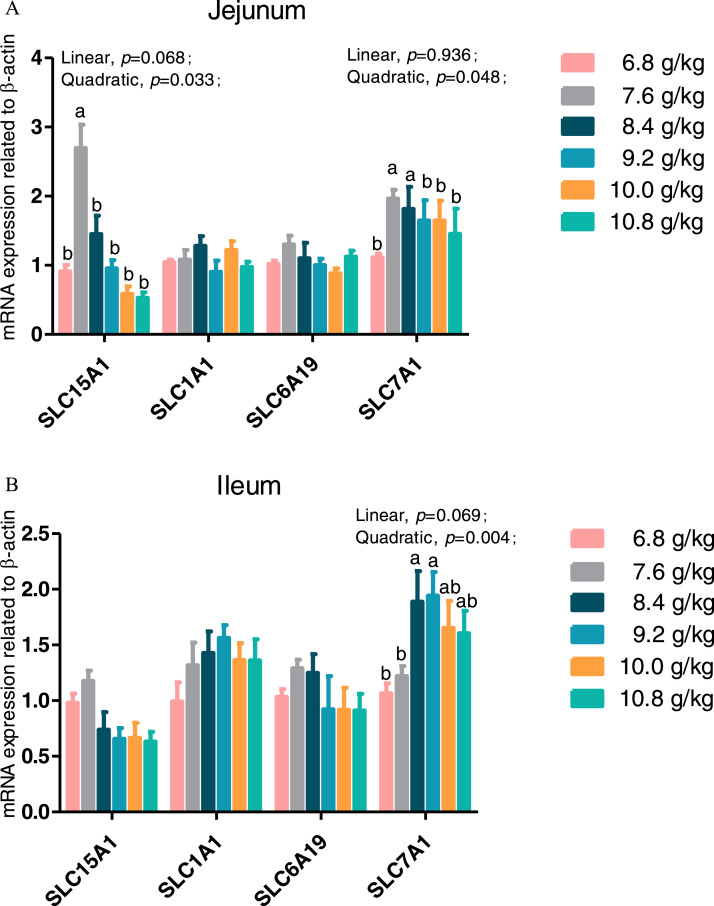

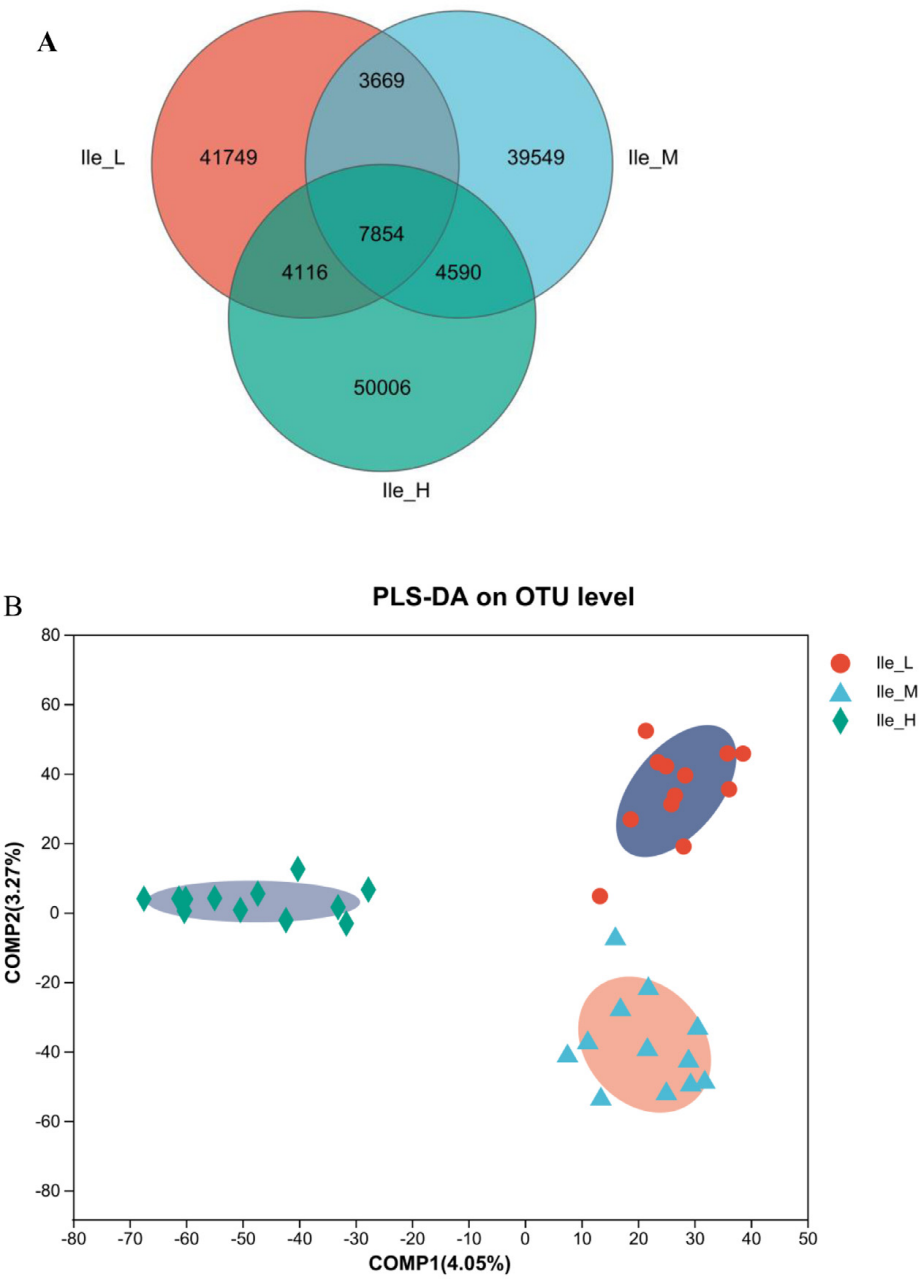

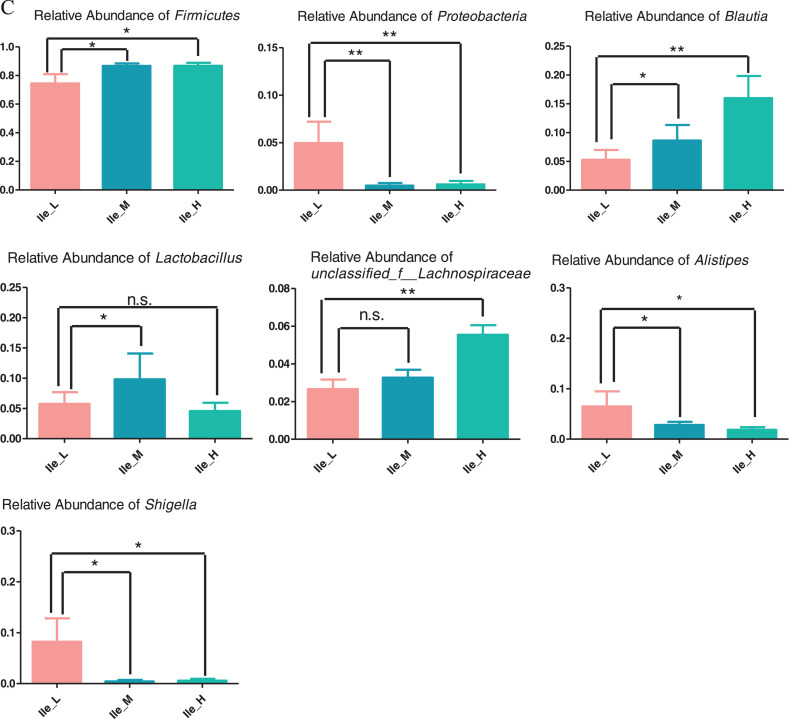

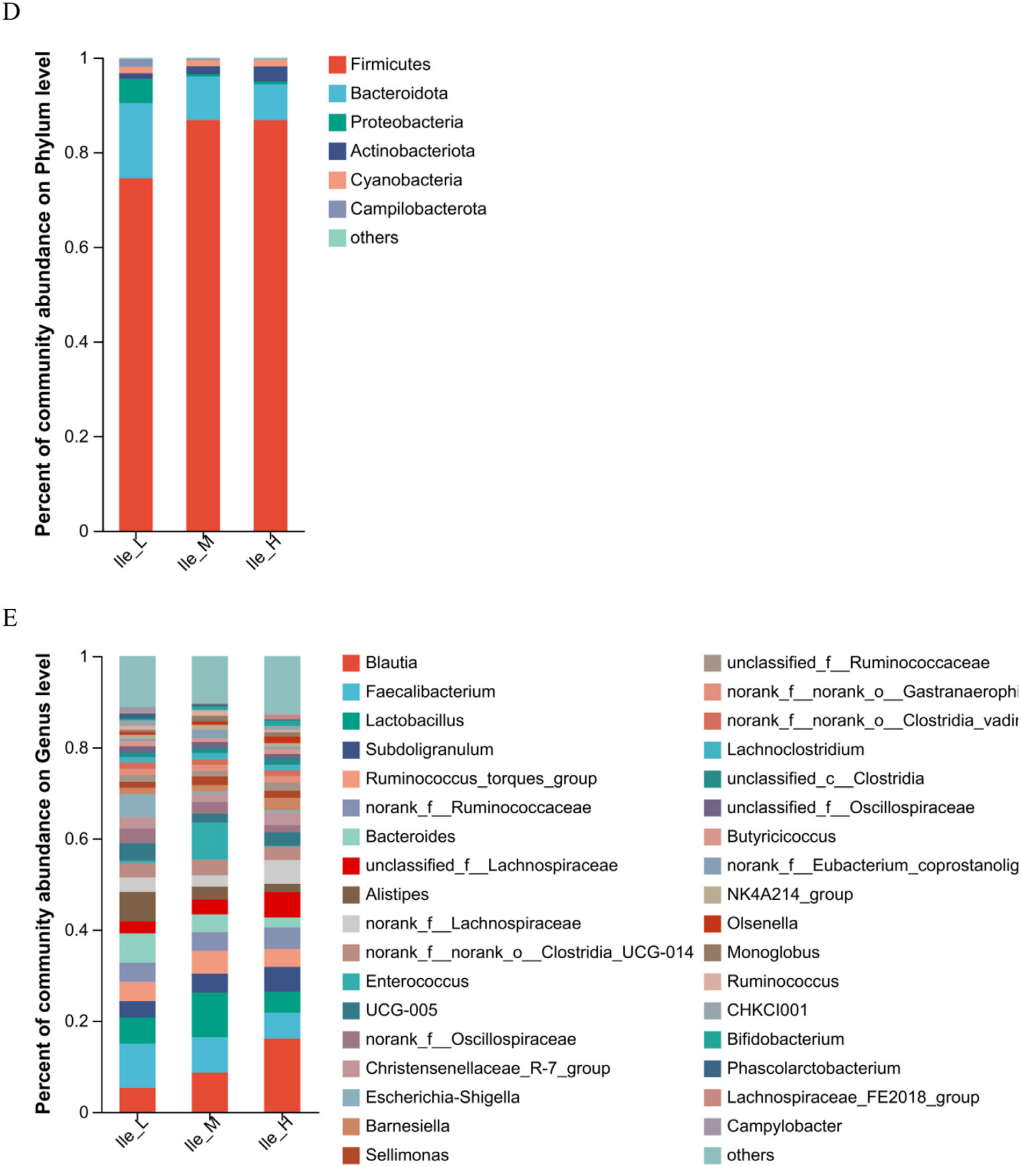
Effect of dietary L-isoleucine levels on the expression genes related to amino acid transporters in jejunum and ileum. *n* = 6. (A) Relative mRNA abundances of solute carrier family 15 member 1 (*SLC15A1*), solute carrier family 1 member 1 (*SLC1A1*), solute carrier family 6 member 19 (*SLC6A19*), and solute carrier family 7 member 1 (*SLC7A1*) in jejunum. (B) Relative mRNA abundances of *SLC15A1, SLC1A1, SLC6A19*, and *SLC7A1* in ileum. Changes in mRNA abundances of *SLC15A1, SLC1A1, SLC6A19*, and *SLC7A1* were normalized to β-actin and expressed relative to the 6.8 g/kg L-isoleucine. Data are means ± SE, *n* = 6. Means with different letters (superscripts a, b) differ (*P* < 0.05). Effect of dietary L-isoleucine on microbiome composition in the cecum of yellow-feathered chickens (*n* = 12). (A) Venn diagram depicting unique and shared OTU dietary groups. (B) Partial least squares discriminant analysis (PLS-DA); (C) Relative abundance of *Firmicute* (at the phylum level), *Proteobacteria* (at the phylum level), *Blautia* (at the genus level), *Lactobacillus* (at the genus level), *unclassified_Lachnospiraceae* (at the genus level), *Alistipes* (at the genus level), and *Enterococcus* (at the genus level); (D) Relative abundance of top 6 phyla in each group; (E) Relative abundance of top 35 genus in each group; Ile_L = chickens received a basal diet with 6.8 g/kg isoleucine; Ile_M = chickens received an experimental diet with 9.2 g/kg isoleucine; Ile_H = chickens received an experimental diet with 10.8 g/kg isoleucine. Data in B were analyzed with unpaired *t* test, * means *P* < 0.05, ** means *P* < 0.01, ns means not significant.

As is illustrated in Figure 3, dietary Ile supplementation increased jejunal expression of peptide transporter by solute carrier family 15 member 1 (***SLC15A1***) in a linear (*P* = 0.068) and quadratic (*P* < 0.05) manner. With increased dietary Ile, there were increasing trends in jejunal and ileal expression of solute carrier family 7 member 1 (***SLC7A1***) (*P* = 0.004). However, there were no changes in gene expression of solute carrier family 1 member 1 (***SLC1A1***) and solute carrier family 6 member 19 (***SLC6A19***) in response to the dietary level of Ile from 6.8 to 10.8 g/kg (*P* > 0.05).

### Gut Microbiome

In total, 151,533 OTU from 36 cecal samples (12 samples each from the low, medium, and high Ile treatments) were identified and each sample had 4,209 OTU on average. Venn diagrams are shown in Figure 3A, and there were 41,749, 39,549, and 50,006 unique OTU identified in Ile_L (6.8 g Ile/kg), Ile_M (9.2 g Ile/kg), and Ile_H (10.8 g Ile/kg), respectively, with 7,854 OTU being shared. There were no significant differences (*P* > 0.05) in alpha diversity including observed species, Chao1, ACE, Shannon, Simpson, and Coverage with dietary Ile content (Table S2). PLS-DA revealed a distinct clustering of microbiota composition for each group (Figure 3B).

As shown in Figure 3C and D, at the phylum level, dietary Ile supplementation increased the relative abundance of cecal Firmicutes (*P* < 0.05) and tended to have lower relative abundance of cecal Proteobacteria (*P* < 0.05). At the genus level, the relative abundance of cecal *Blautia, Lactobacillus*, and *unclassified_Lachnospiraceae* genera were significantly increased in chickens fed diets supplemented with dietary Ile (*P* < 0.05, Figure 3C and E), compared with birds fed the Ile-deficient diet. Diet supplemented with Ile significantly decreased the relative abundance of ileal *Alistipes* and *Shigella* genera (*P* < 0.05, Figure 3C and E). At the species level, birds fed Ile-supplemented diets had increased relative abundance of cecal *unclassified_g_Blautia* and *unclassified_f_Lachnospiraceae* species (*P* < 0.05, Figure S1) compared with birds fed the Ile-deficient diet.

### Estimation of the Dietary Requirements for Isoleucine

Dietary Ile requirements of medium-growing yellow-feathered chickens between 1 and 30 d of age, estimated by quadratic and 2-slope broken line regression analyses, are present in Table 5. Based on quadratic regression, the dietary Ile requirements were 8.8, 8.8, and 8.1 g/kg, respectively. 2-slope broken line regressions were 7.6, 7.8, and 8.8 g/kg, respectively.

**Table 5:** Estimations of dietary L-isoleucine requirements based on nonlinear regressions of average daily gain and feed conversion ratio on dietary isoleucine concentrations.

Dependent variables	Model^1^	Regression equation^2^	*R*^2^	*P*	Dietary Ile Requirement, g/kg^3^	Dietary Ile/Lys
Average daily gain	QP	*Y* = 3.044 + 3.904*X* − 0.210*X*^2^	0.731	0.018	8.8	73/100
	Two-slope BL	*Y* = 21.07 − 1.853 × (7.6 − *X*) (*x* ≤ 7.6) *Y* = 21.07 − 0.065 × (*X* − 7.6) (*x* > 7.6)	0.980	<0.001	7.6	63/100
Feed conversion ratio	QP	*Y* = 3.082 − 0.270*X* + 0.015*X^2^*	0.852	0.012	8.8	73/100
	Two-slope BL	*Y* = 1.831 + 0.088 × (7.8 − *X*) (*x* ≤ 7.8) *Y* = 1.831 + 0.004 × (*X* − 7.8) (*x* > 7.8)	0.673	0.007	7.8	65/100
Plasma UA content	QP	*Y* = 330.3 − 67.421*X* + 3.942*X^2^*	0.926	0.003	8.1	68/100
	Two-slope BL	*Y* = 37.57 + 8.056 × (8.8 − *X*) (*x* ≤ 8.8) *Y* = 37.57 + 11.981 × (*X* − 8.8) (*x* > 8.8)	0.973	0.015	8.8	73/100

1 QP = quadratic polynomial; BL = broken line; UA = uric acid.

2 *Y* is the dependent variable and *X* the dietary Ile concentration, g/kg.

3 Dietary Ile requirement = the optimal dietary Ile concentration, g/kg.

## DISCUSSION

Dietary Ile was shown here to have positive effects on ADG, FCR, and 30-day BW in medium-growing yellow-feathered chickens. Chickens fed Ile-deficient diets (5.1 g Ile/kg) had poorer weight gain and FCR during 18 to 30 d of age. Adding Ile at 0.5, 1.0, 1.5, or 2.0 g/kg additional Ile to chicken's diet that contained 5.1 g Ile/kg during grower phase improved growth rate, feed consumption, and FCR (Kidd et al., 2004). Additionally, increasing Ile from 4.2 to 6.6 g/kg in broilers’ diets during the finisher phase improved ADFI, FCR, and final BW (Hale et al., 2004b), whereas increasing Ile levels from 5.8 to 6.6 g/kg in broiler diets during the finisher phase did not change market weight, but only improve FCR (Corzo et al., 2008). The needs for dietary Ile level have been inconsistently determined in previous research using different birds, strains, diets with varied Ile contents. The present study determined that 8.8 g/kg of total dietary Ile was the optimum level for medium-growing yellow-feathered chickens (1–30 d) to get the highest increase in BW.

Ma et al. (2023) found that adding Ile in the broiler diet from hatch to 42-day decreased serum TG, TC, and body fat accumulation. They also observed that Ile regulated insulin secretion, which could be reflected in blood concentrations of GLU. Liu et al. (2023) reported that diet supplemented with Ile decreased serum concentrations of UA, GLU, and TC in Arbor Acres broilers at 42 d of age. Ullah et al. (2022) observed that increasing dietary levels of Ile from 6.6 to 8.6 g/kg in laying hens reduced serum levels of GLU. By contrast, blood TC and GLU were not altered by feeding laying hens diets supplemental with various levels of Ile, but GLU concentrations increased (Dong et al., 2016). In the experiment, yellow-feathered chicks fed diets containing 7.6 to 8.4 g Ile/kg had the highest plasma concentration of ALB and the lowest of UA. However, there were no change on plasma contents of TG, TC, and GLU with diets supplemented with Ile. These findings indicated that dietary Ile supplementation enhanced protein anabolism and suppressed protein degradation. The protein status may explain the increased ADG in those chickens, whereas the effect of Ile on lipid metabolism may not obvious at brooding phase and genetic back ground, type of production and/or experimental period may explain GLU and TC findings were not consistent with previous findings (Rajman et al., 2006; Xie et al., 2015; Liu et al., 2023). Cellular damage is reliably monitored with circulating CK, GPT, GOT, and LDH activities (Jiang et al., 2019). Of these, only GOT concentration decreased by dietary Ile supplementation in yellow-feathered chickens. In line with previous studies, increasing Ile level, or valine, or threonine did not affect blood CK, GPT, GOT, and LDH (Azzam et al., 2015; Dong et al., 2016; Jiang et al., 2019). These findings indicated that dietary Ile supplementation maintained normal hepatic function of yellow-feathered chickens. It has been stated that BCAA-modulated immunity in chickens and other animals (Konashi et al., 2007; Li et al., 2007). Similar findings were reported in chickens fed diets containing Ile ranging from 4.2 to 8.2 g/kg did not show changes in the weight of immune organs like the spleen, thymus, and Bursa (Hale et al., 2004a). This result could be due to concentration of Ile in the basal diet was insufficient to induce changes in immune organs.

One of the main signaling pathways activated by amino acids is the MTOR, which participates in protein synthesis. The RPS6KB1 and EIF4EBP1 are 2 of the best-characterized downstream targets of MTOR regulating mRNA translation by phosphorylation (Deng et al., 2014). The major proteolytic system involves lysosomal, Ca^2+^-dependent calpains, and ubiquitin/proteasome-dependent systems. The ATP-dependent ubiquitin/proteasome system is the most important proteolytic pathway, while cathepsin B is a cysteine protease primarily involved in degradation of lysosomal proteins (Yan and Sloane, 2003). Moreover, MAFbx and MuRF1 are 2 important ubiquitin ligases which predominantly mediate structural muscle protein degradation (Bodine and Baehr, 2014). Dietary Ile supplementation likely influenced protein turnover by means of improved protein biosynthesis via enhancing gene expression of *RPS6KB1* and *EIF4EBP1* in jejunum and suppressed protein degradation via decreasing mRNA expression of *MuRF1* and *CC2* in jejunum and ileum of chickens. Kim et al. (2022) suggested that BCAA such as Ile, leucine and valine promoted protein production and inhibited protein breakdown in poultry. Similarly, Chang et al. (2015) reported that dietary supplementation with BCAA such as leucine augmented protein anabolism by improving intestinal mRNA expression of *MTOR*, p70 *RPS6KB1*, and *EIF4EBP1* in chickens, which impacted growth of intestinal epithelial cells and reflected positively villus height. Dietary Ile supplementation in fish improved growth performance as a result of boosting protein synthesis by enhancing mRNA expression of *TOR, RPS6KB1*, and *EIF4EBP1* in liver, intestine, and muscles (Zhou et al., 2020; Jiang et al., 2021). Liu et al. (2021a) reported that Ile increased muscle mass through myogenesis and intramyocellular lipid deposition. The mTOR-S6K1 signaling pathway, modulated by high doses of Ile, regulates pancreatic tissue amylase, trypsin, and chymotrypsin excretion (Cao et al., 2019). As a critical regulator of metabolic health, a low-Ile diet reprograms liver and adipose metabolism to enhance hepatic insulin sensitivity and ketogenesis, as well as increasing energy expenditure by activating the fibroblast growth factor 21-uncopling protein 1 axis (Yu et al., 2021). Thus, the current results combined with the previous studies suggest that Ile is involved in upregulating the mRNA expression of *RPS6KB1* and *EIF4EBP1*, accompanied by inhibiting the mRNA expression of *CC2* when modulating protein synthesis in the intestine.

Intestinal expression of genes encoding peptide and amino acid transporters can shed light on the amino acid absorption mechanism (Osmanyan et al., 2018). One of the important intestine transporters, SLC15A1, is thought to be responsible for absorbing the majority of dipeptides and tripeptides (Spanier, 2014). Previous research demonstrated that the mRNA expression of *SLC15A1* can be influenced by certain amino acids in feed, such as threonine, leucine, and glutamine (Jiang et al., 2019; Ji et al., 2023; Reicher et al., 2022). The SLC6A19 transporter, also known B^0^AT, is an Na^+^-dependent neutral amino acid transporter in the brush border of the intestine, where it plays a role in the absorption of neutral and cationic amino acids (Margheritis et al., 2016). Amino acids like aspartate and glutamate, which are anionic, are particularly well preferred by SLC1A1, which also referred to as excitatory amino acid transporter 3 (Hundal and Taylor, 2009). The jejunal and ileal expression of *SLC6A19* and *SLC1A1* in Chinese yellow-feathered chickens was not significantly affected by dietary Ile supplementation, whereas that of *SLC7A1* increased significantly in the jejunum and ileum, which thought to modulate absorption of nutrients in chickens. The SLC7A1 also referred to as Na^+^-independent cationic amino acid transporter 1 (**CAT1**) is responsible for nonepithelial cationic amino acid transport and is widely expressed including the intestine (Bröer and Fairweather, 2019). There are very few previous studies in poultry examining the impact of Ile on amino acid transporters. Cervantes-Ramírez et al. (2013) found, however, that leucine and Ile alone or in combination increased jejunal expression of *CAT1* in pigs. Similarly, Zhang et al. (2013) documented that jejunal *CAT1* transcripts were more abundant in piglets fed the low protein diet supplemented with leucine, Ile, and valine. Consistent with findings in the present study, the higher SLC7A1 transcript levels may be due to an improvement in nutrient digestibility and the transporter proteins expression in the apical membrane of small intestine enterocytes (Bröer and Gauthier-Coles, 2022).

The intestinal microbiota is a complex community of microorganisms inhabiting the gastrointestinal tract that is crucial for host's intestinal function and health. Intestinal permeability, nutritional digestion, and metabolism, as well as immunological responses can vary as a result of changes in the composition and function of the gut microbiota (Fan and Pedersen, 2021). In the present study, PLS-DA showed that the bacteria in cecum formed a distinct cluster and were not connected to each other. According to the results of ANOSIM analysis, the cecal microbiota of yellow-feathered chickens with moderate or high Ile supplementation (Ile_M and Ile_H) were similar, and they were different from those of Ile-deficient birds (Ile_L). At the phylum level, compared with the Ile_L, there were more Firmicutes, Firmicutes: Proteobacteria (**F/G**) and fewer Proteobacteria in cecal digesta with Ile supplementation. A relative increase in Firmicutes would be beneficial for gut health. Firmicutes are dominant hydrolyzed bacteria in the intestinal tract which are responsible for the production of lactic acid and carbon dioxide through the breakdown of organic sugars during fermentation (Sun et al., 2022). The phylum Firmicutes includes the majority of bacteria that produce short-chain fatty acids (**SCFAs**), more Firmicutes means a stronger gut barrier and less inflammation (Huang et al., 2018). Alterations to the F/B ratio have been linked to a variety of diseases and dysfunctions because of their role in homeostasis maintenance. Variations in the F/B ratios, either higher or lower, have been linked to obesity and inflammatory bowel disease, respectively (Stojanov et al., 2020). Proteobacteria, one of the most abundant phyla, comprising a wide variety of pathogenic genera, such as *Escherichia coli, Shigella*, and *Salmonella*, are often overrepresented in several intestinal diseases, mostly with an inflammatory phenotype (Rizzatti et al., 2017). At the genus level, dietary Ile significantly raised relative abundance of *Blautia, Lactobacillus*, and *unclassified_Lachnospiraceae*, whereas decreasing that of *Alistipes* and *Shigella*. The genus *Blautia* contains anaerobic bacteria commonly found in the feces and intestines of mammals having probiotic properties. This genus contributes to the biotransformation process, as well as the management of host health and alleviation of metabolic syndrome (Liu et al., 2021b). *Lactobacillus*, the largest genus of lactic acid bacteria, possess various probiotic characteristics important for resistance to gastrointestinal tract infections and diseases (Masood et al., 2010). Ma et al. (2020) reported that the ratio of F/B was decreased after Ile supplementation in a high fat diet. In the gut of Jian carp, Zhao et al. (2014) showed that Ile stimulated the development of *Lactobacillus* and *Bacillus*, while suppressing the growth of *Aeromonas* and *Escherichia*. In vitro, Ile starvation decreased the growth of *Lactococcus lactis* (Dressaire et al., 2011). As early colonizer, *Lachnospiraceae* belong to the core of gut microbiota, are main producers of SCFAs, of which certain SCFAs such as butyrate are thought to improve weight gain in chickens challenged with *E. maxima* infection (Hansen et al., 2021). In summary, in yellow-feathered chickens, dietary Ile was exhibited to modulate the composition of gut microbiota and likely enhanced growth performance.

## CONCLUSIONS

In conclusion, dietary Ile levels significantly affected growth performance and modulated gut microbiota structure in medium-growing yellow-feathered broilers. The optimal Ile levels from quadratic regression (and broken line analyses) for maximizing ADG, FCR, and plasma UA were 8.8 (7.6), 8.8 (7.8), and 8.1 (8.8) g/kg, respectively. The appropriate level of dietary Ile can upregulate the expression of intestinal amino acid transporters and protein synthesis-related protein kinase genes and concomitantly inhibit the expression of proteolytic-related cathepsin genes.

## Appendix Supplementary materials

Image, application 1

## Footnotes

Supplementary material associated with this article can be found in the online version at doi:10.1016/j.psj.2023.102774.
