# Supplementary material for: Dietary isoleucine supplementation enhances growth performance, modulates the expression of genes related to amino acid transporters and protein metabolism, and gut microbiota in yellow-feathered chickens
Source: Poult Sci. 2023 May 9;102(8):102774. doi: 10.1016/j.psj.2023.102774 (PMC10276271; doi:10.1016/j.psj.2023.102774)
Supplement: Supplementary file 1 [file mmc1.docx]

**Table S1.** Effects of dietary *L*- isoleucine on digestive and immune organ indexes of yellow-feathered chickens at 30 days of age.^1^

| Indices^2^ | Dietary Ile content, g/kg | | | | |  | SEM | *P*-value | | |
| --- | --- | --- | --- | --- | --- | --- | --- | --- | --- | --- |
|  | 6.8 | 7.6 | 8.4 | 9.2 | 10.0 | 10.8 |  | ANOVA | Linear | Quadratic |
| Digestive organ |  |  |  |  |  |  |  |  |  |  |
| Proventriculus | 0.63 | 0.56 | 0.63 | 0.59 | 0.56 | 0.63 | 0.024 | 0.101 | 0.818 | 0.174 |
| Gizzard | 2.95 | 2.76 | 3.03 | 3.20 | 2.78 | 3.13 | 0.126 | 0.174 | 0.300 | 0.936 |
| Pancreas | 0.36 | 0.36 | 0.37 | 0.37 | 0.35 | 0.37 | 0.019 | 0.264 | 0.772 | 0.985 |
| Duodenum | 1.40 | 1.25 | 1.27 | 1.36 | 1.30 | 1.24 | 0.068 | 0.468 | 0.215 | 0.948 |
| Jejunum | 2.45 | 2.20 | 2.25 | 2.36 | 2.30 | 2.22 | 0.069 | 0.783 | 0.514 | 0.710 |
| Ileum | 1.71 | 1.62 | 1.48 | 1.69 | 1.59 | 1.55 | 0.080 | 0.346 | 0.229 | 0.512 |
| Immune organ |  |  |  |  |  |  |  |  |  |  |
| Spleen | 0.15 | 0.17 | 0.20 | 0.20 | 0.16 | 0.18 | 0.022 | 0.344 | 0.172 | 0.498 |
| Thymus | 0.55 | 0.62 | 0.58 | 0.50 | 0.53 | 0.52 | 0.044 | 0.598 | 0.181 | 0.911 |
| Bursa | 0.42 | 0.43 | 0.40 | 0.41 | 0.40 | 0.42 | 0.042 | 0.973 | 0.816 | 0.642 |

^1^Means from 2 birds per pen and 6 replicate pens per diet.

^a-c^Means within a main effect with the same superscripts do not differ significantly (*P* < 0.05).

**Table S2.** Effects of dietary *L*- isoleucine on diversity of bacterial communities in cecal digesta of yellow-feathered chickens at 30 days of age.^1^

| Variable^2^ | Ile_L | Ile_M | Ile_H | SEM | *P*-value |
| --- | --- | --- | --- | --- | --- |
| Species observed | 9711 | 9644 | 11746 | 807.4 | 0.128 |
| Shannon | 7.10 | 7.06 | 7.76 | 0.331 | 0.255 |
| Simpson | 0.016 | 0.022 | 0.014 | 0.011 | 0.502 |
| Chao | 51261 | 47775 | 61589 | 5640.5 | 0.213 |
| ACE | 121461 | 108912 | 147523 | 14920.5 | 0.191 |
| Good’s coverage | 0.75 | 0.76 | 0.71 | 0.026 | 0.294 |

^1^Values are means with the pooled SEM, n = 12.

^2^Ile_L = chickens received a basal diet with 6.8 g/kg isoleucine; Ile_M = chickens received an experimental diet with 9.2 g/kg isoleucine; Ile_H = chickens received an experimental diet with 10.8 g/kg isoleucine.


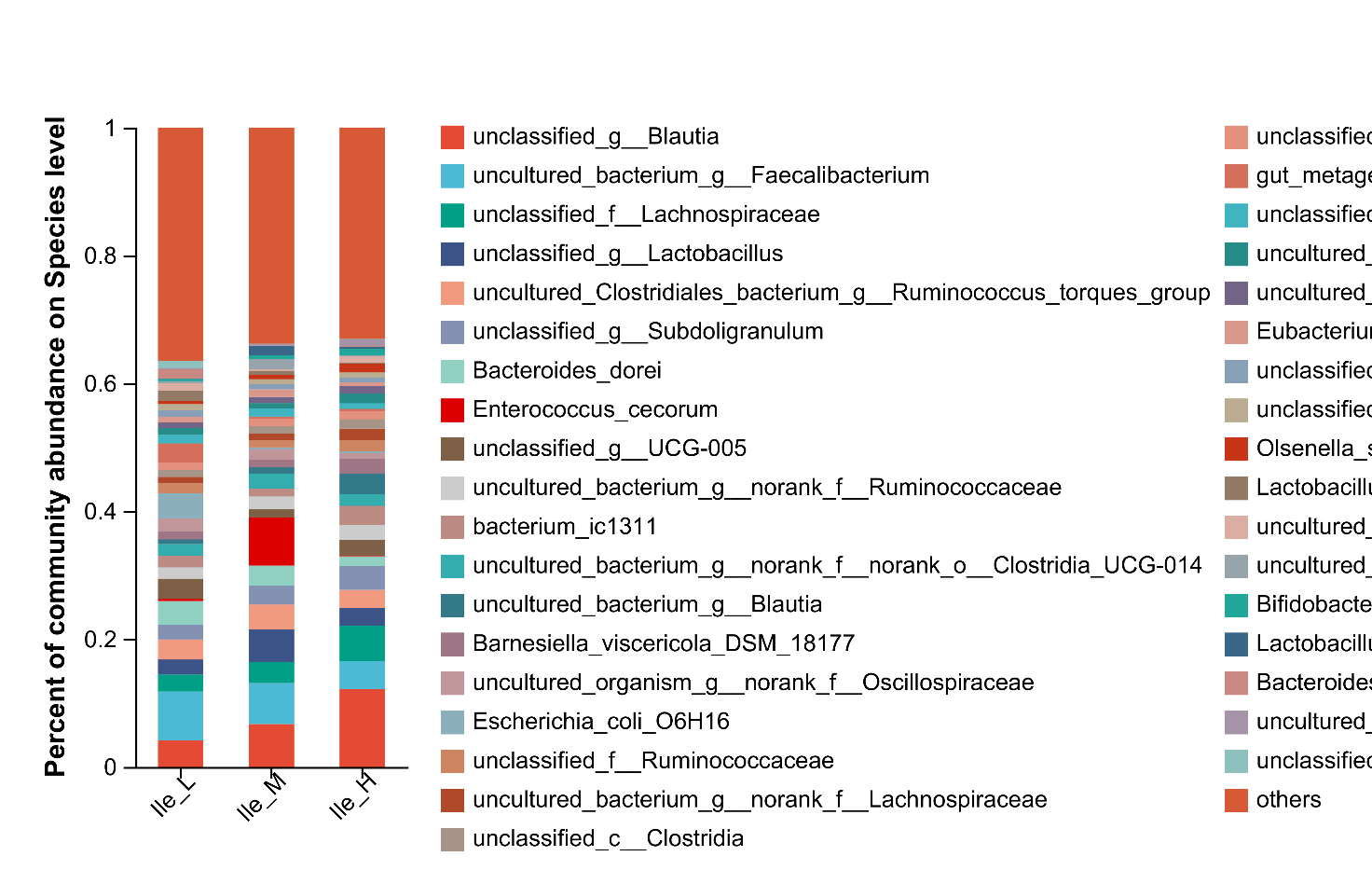


**Figure S1.** Relative abundance of top 35 species level in the cecum of yellow-feathered chickens (n = 12). Ile_L = chickens received a basal diet with 6.8 g/kg isoleucine; Ile_M = chickens received an experimental diet with 9.2 g/kg isoleucine; Ile_H = chickens received an experimental diet with 10.8 g/kg isoleucine.
